# On the Recent Advances in the Theory of Vision

**Published:** 1872-08

**Authors:** H. Helmholtz, F. W. Abbott

**Affiliations:** The Royal Society of London


### Translations.

### *On the recent Advances in the Theory of Vision. By* H. HELMHOLTZ, *(of the Royal Society of London.)*

Translated from *Les Annales d' Oculistique,* By F. W. ABBOTT, M. D.

(*Continued from July No.*)

We have only had to do, up to the present time, with the notion of a superficial field, or with two dimensions, and as its representation was, up to a certain point, analogous to the retinal images, the facts could accord almost equally well with one or the other of the two theories before us. Affairs are very different when we pass to the vision of near objects, for which it is necessary to take account of the depth, or the third dimension: then there is manifested an essential and radical difference, on the one hand between the images which the two retinas receive, and on the other hand between these images and the external world or the accurate notion which we form of it. It is upon this ground that it is necessary to look for the solution of the question which occupies us; and, in truth, for a number of years the theory of the perception of relief, and that of binocular vision, to which we principally owe this perception, have been a field of battle upon which have striven the most diverse researches and opinions. We see, indeed, from what precedes, that the questions here presented are of the most capital importance since the whole range of human knowledge is interested in their solution.

Each one of our eyes receives upon its retina an image which extends only upon the surface. Whatever be the disposition which we wish to assign to the nervous conduit, no hypothesis enables us to represent in the brain the entirety of the two retinal images by any thing else than by a superficial image. Now the two superficial images united give us the notion of a material image As when we were concerned with colors, we here find a greater quantity in the external world than in sensation; but this time the notion to which we attain is inferior in no respect to the multiplicity of the form of the objects. It is important to remark that, the notion of the third dimension is as lively, as exact, and as immediate in us, as that of the two other. When we jump from one rock to another, our safety depends just as much upon the appreciation of the distance to be crossed as of the direction in which we must jump. This second appreciation is relative only to the position in the visual field, and experience teaches us that it is made neither more quickly nor more accurately than that of distance.

How is the notion of distance produced? Let us commence by examining the facts.

We must remark at first, that up to a certain point we distinguish the relief and the distance of objects even when we use only one eye, and do not move the head. The circumstances which facilitate this appreciation in us differ very little from those by which painters profit to produce the illusion of relief and of distance, to which we attach so much value. Let us see how landscapists proceed. They prefer the moments when the sun is near the horizon, on account of the vigorous shadows which then best bring out the form of objects; they like also a light mist which causes the back ground to recede. People and animals, objects whose size is known to us, serve them in giving us standards as to the size and apparent distance of the other objects represented: the products of human industry, such as houses have the effect of giving with accuracy the position of the horizontal.

Buildings, machines, utensils are the objects a complete representation of which is best given us by an accurate perspective. We know that these objects are bounded on almost every side by planes which intersect at right angles, or by curved or spherical surfaces. This knowledge enables us to fill out the information furnished by the sketch. The symmetrical structure of the human body and of animals facilitates equally the comprehension of the respective representations of these objects.

When we have to do, on the contrary, with the representing of bodies of unknown and very irregular form, such as rocks, blocks of ice &c., we see the most skillful art of the painter frustrated; much more, the rigorously faithful representation which the photograph gives us of these objects shows us often only an unintelligible crowd of bright or dark spots. The contemplation of the objects themselves enables us, on the contrary, to recognize at once their exact form.

It is to one of the most illustrious of the painters, and almost as great a physicist as artist, Leonardo deVinci, that the honor belongs of having clearly explained in what the best painting is necessarily inferior to the contemplation of the real objects. In his *Trattato della Pittura*, he remarked that our two eyes do not cause us to see the external world under an absolutely identical aspect. Each eye receives upon its retina a perspective representation of the surrounding objects; but, as these two organs are situated at some distance the one from the other, the difference of point of view involves a slight difference between the perspective representations formed in either eye. If, holding my index finger vertically before my face, I close each eye alternately, the finger hides from me alternately upon the wall, a part situated now more to the right, now more to the left. If I hold the right hand vertically before me, the thumb being nearer me than the rest of the hand, according as I use the right eye or the left eye I perceive a greater part of the back or of the palm of this hand, and things occur in an analogous manner whenever we look at objects whose different parts are at different distances from our eyes. But if we see in a picture a hand placed as I have just described, both eyes see exactly the same representation; one sees as much as the other of either surface of the hand. So, while the real objects give us different images to each eye, a painting gives them the same images. Here is a difference of impression which the skill of the artist cannot do away with.

The importance of binocular vision for the perception of relief has been illustrated in a very remarkable manner by the stereoscope which Wheatstone invented. Every one knows of this instrument and the remarkable illusion which it is designed to produce. The objects represented appear in it with a relief as indubitable as though we had the real objects before our eyes. This result is obtained by presenting to the two eyes images a little different in perspective, and such that each one receives an image like that which the real object would give. If the images employed are well executed, if, for example, they are two photographs taken from slightly different points of view, the impression produced is, minus color, absolutely the same as though we were in the presence of the reality.

Those who are sufficiently masters of the movements of their eyes have no need of an instrument to fuse two stereoscopic images, and obtain the impression of relief; it is sufficient for them to direct their gaze so as to fix at the same time the corresponding points of the two images. But, for the majority of observers, it is easier to employ instruments whose effect is to cause the images to appear in the same place.

In the instrument such as Wheatstone constructed at first, each eye looks in a mirror placed obliquely as it regards its visual line, and the two designs were laterally disposed so that their reflected images occupied the same position in space. Each eye perceived one of these two images.

The stereoscope with prisms, or Brewster's, which gives less clear images is more widely known on account of its greater handiness. Both designs are upon the same card, which is placed at the bottom of the box, which is divided into two parts by a partition. In front are two slightly prismatic convex glasses, whose effect is to remove and slightly to enlarge the images, while displacing them laterally, so that the observer thinks he sees them at the same place; each eye sees the image which is designed for it, and both appear to be in the middle of the box.

It is particularly when the other elements which generally aid us in recognizing the form of objects have failed us, that the stereoscopic illusion presents itself in the most striking manner; I will note geometrical figures in simple outline, such as representations of crystals, or better still the pictures of very irregular objects, above all when, these objects being more or less transparent, their shadows are not presented in a manner familiar to us. It is in this manner that each one of the two stereoscopic photographs of glaciers often presents to the eye only the aspect of an irregular marbling, while by uniting the two in the instrument we seem to see the crevasses in the ice, the plays of light which penetrate it and even the glitter of the slippery surfaces of the glacier.

More than once monuments, cities, landscapes, whose stereoscopic representations I had seen, have made no impression of novelty upon me, a thing which never happened to me when I had seen only sketches or pictures, which can never produce a sensation comparable to that which is given by the reality.

The exactness of stereoscopic vision is astonishing. To give an example let us cite an application which Dove has proposed. If we put into a stereoscope two proofs obtained from the same composition of types or the same engraved plate, these images, perfectly equal, will evidently give a perfectly plane resulting image. Now, human skill is not sufficient to imitate the characters or the lines of an engraved plate with such accuracy that, in placing simultaneously under the stereoscope two proofs, obtained from two plates, certain letters or certain lines will not appear to stand out from the paper. This is the easiest means of recognizing counterfeit bills; we place a good bill and the suspected bill in the instrument together, and we see whether in the resulting image, all the lines appear to be in the same plane.

This experiment is important as it regards the theory of vision on account of the striking manner in which it shows with what delicacy we perceive the differences of relief brought out by the difference between our retinal images.

We must now try to discover how two retinal images, both superficial, can, by their union, produce in us a corporeal notion,—that of a body with three dimensions.

Let us determine, in the first place, that both of the superficial images furnished by our eyes are really perceived. When I look at the wall of my room and interpose my finger held vertically, this finger, as we have said before, hides from each of our eyes a different part of the wall, then when the wall is seen single the finger appears double.

Since, in ordinary vision, we are only concerned in distinguishing the objects themselves these double images escape our notice except in very peculiar cases. To see them it is necessary to look at the field of vision in a very different manner; we must do as a sketcher does who wishes to reproduce it. The artist tries to forget the true form, size, and distance of the objects which he wishes to represent. He forces himeelf to see them simply as they appear in the superficial field of vision, in order to reproduce them upon the superficies of his paper. It would be natural to think that such is the original and most simple manner in which the vision can be used; so the majority of physiologists have up to this time considered it as being the form in which the notion is immediately imposed upon the sensation, and they have considered the vision of the relief as a result of study, as a representation experimentally acquired, and resulting from a secondary manner of using the vision. But it suffices to have sketched a little, to know how much more difficult it is to appreciate and to measure the apparent form of objects in the visual field, than to recognize their true form and size. The notion of the real object, of which the sketcher cannot divest himself, constitutes in truth the greatest difficulty in sketching from nature.

Let us then look at the field of vision after the manner of sketchers, keeping both eyes open; when we turn our attention to the objects which cover its superficies, the differences between the two retinal images can no longer escape us: we see double the objects situated on this side of and beyond the point of fixation, provided that they are not so far removed from the line of sight as not to be distinctly perceived. At first we notice only the widely separated double images, but very soon, by practice, we can distinguish even those which are near each other. In like manner if I hold a finger vertically before me, as has been said before, this finger hides a different part of the wall from each of my eyes; if I look at a point of the wall, the wall is seen single and as the finger projects itself upon two different points of its surface, it must be that the finger should be seen double.

All these phenomena, and other analogous ones, such as those presented by the position of the double images of an object seen binocularly, may be embraced under a simple law, which has been formulated by Jean Mueller. To each *point* of one retina corresponds upon the other a *corresponding point*. In general, in the superficial field of vision common to both eyes, the images which appertain to corresponding points coincide; those which appertain to points not corresponding do not coincide. Or, what is about the same, the points of the two retinas situated at the same distance to the right or the left, above or below the point of fixation are corresponding.

We have seen before that the naturalistic theory necessarily supposes a perfect fusion of the sensations transmitted by the *corresponding* points, which Mueller also called *identical.*

This hypothesis takes on a more palpable form when we join to it the anatomical hypothesis, in accordance with which the corresponding fibers blend two by two, either at the point of crossing of the optic nerves, or in the brain. We hasten to add that, although indicating the possibility of such a mechanical explanation, Jean Muller by no means vouched for its accuracy. He considered his law of identical points as being the expression of the facts, and he insisted only upon the identy which he believed that he recognized between the localizations of the sensations received by these points.

But here this difficulty presents itself, that every time the double images are susceptible of being fused into one single notion, their isolated perception has relatively little accuracy, which contrasts with the extreme precision of which the appreciation of relief is susceptible, as Dore has demonstrated. Nevertheless, it is solely upon this difference between the two images, from which double images result, that the vision of relief rests. A very little difference between two stereoscopic images suffices to produce in us the impression of a convex surface, while a difference from twenty to thirty times greater would be necessary for the double images to become perceptible, even to the most experienced observer.

Moreover, other and different circumstances render the perception of double images sometimes more easy, sometimes more difficult. In this connection the notion of relief acts in a very remarkable manner—the more evident it becomes, the more difficult it is to see the double images. It is for this reason that they are perceived less in the presence of real objects than when we look at stereoscopic images. The perception of them becomes more easy, on the contrary, either when the corresponding lines of the two designs differ in intensity or in coloring, or if we add lines and points which occupy exactly corresponding positions, which brings out the failure of the correspondence of the neighboring lines. We could not explain the influence of all these circumstances if the unity of the localization was founded upon an anatomical communication of the nervous conduits.

Another difficulty, raised by the discovery of the stereoscope, was to explain how the perception of relief results from the difference between the two retinal images. Brucker first remarked a series of facts which appear to render the stereoscopic phenomena reconcilable with the theory of the innate identities of the retinas. If we observe ourselves while we look at stereoscopic images, or analogous objects, we ascertain that our eyes run along the different contours in such a manner that, while the point of fixation is seen single, the other parts may very easily seem doubled. Now, our attention being habitually concentrated upon the point of fixation, we take so little notice of the double images, that this phenomenon is often an unexpected discovery to those whose attention we call to it. As the act of following the contours of an object demands irregular movements, accompanied with variations of convergence as much greater as the distance between the different points of the object is more variable, we may conceive that the consciousness of these movements may produce the notion of the difference of the distances of the lines examined.

It is incontestible, in truth, that these movements of the sight give, much better than the fixation of one single point, the notion of relief represented by a linear stereoscopic design, only because these movements cause us to apply successively to the different points of the figure direct vision, which is much more clear than indirect.

The hypothesis of Brucker, according to which the perception of relief is obtained solely by the movements of the eyes, cannot be sustained since the experiments of Dove, according to which the stereoscopic illusion persists while employing the illumination of the electric spark, whose duration does not reach the four-millionth part of a second. In so short a time moving, terrestrial bodies move so little that, whatever be the velocity of their motion, they seem to remain absolutely immobile. During the duration of the electric spark there cannot be produced, therefore, any appreciable movement of the eyes; nevertheless, the illusion of stereoscopic relief is produced perfectly well.

The phenomenon of stereoscopic luster, also discovered by Dore, demonstrates that there does not exist between the sensations of the two eyes such a fusion as is demanded by the anatomical hypothesis. When a surface white in one of the stereoscopic images is black in the other, its binocular image takes a peculiar shine or luster, even if the paper is perfectly dull. Stereoscopic designs of crystalline forms have often been executed, one of the images being drawn in white on a black ground, the other in black upon a white ground; in the stereoscope we then seem to see a crystal of shining graphite. In accordance with the same principle, a photograph often reproduces, in the stereoscope, the effect of a sheet of sparkling water, the varnished leaves of certain plants, &c.

This is the explanation of this phenomenon. An unpolished surface, such as a white paper presents, reflects light with an equal intensity in all directions. It is for this reason that this surface appears equally luminous to us, whatever may be the point of view chosen; it also appears equally luminous to either eye. A polished surface, on the contrary, besides the light which it diffuses equally in all directions, gives reflections in certain determined directions. Now, it may be that only one of the eyes receives this reflected light; then the reflecting surface appears much more luminous to that eye, and, as this is an effect which only polished bodies can produce, the presence of a difference of light in the stereoscope gives us the impression of luster.

If the impressions furnished by the retinal images were fused together, the union of black and white would make gray. This circumstance, that black and white stereoscopically combined give us the impression of lustre—a sensual impression which no gray surface can give—is sufficient to prove that the sensations of the two retinas *do not become fused.*

The instantaneous illumination by the electric spark gives the impression of luster perfectly. This proves that this impression does not rest on an alternation of sensation between the two eyes, nor upon what it called the antagonism of the retinas.

More, also, not only are the sensations of both eyes not fused, but we also distinguish each from the other the sensations of either eye.

Finally, the phenomena which occur when we hold before the two eyes two images which are not susceptible of being united into the notion of one object, present a peculiar interest. When, for example, one of the eyes looks at a printed page, and the other at an engraving, the *antagonism* or *conflict* of the visual fields prevents the two images from being simply and purely superimposed; one or the other always predominates by spots or places. If both designs are equally clear, the places where the one or the other predominates generally move after a few seconds; but if one of the images presents in any part a uniform white or a black ground, to which some very clear contours correspond in the other, then these last generally predominate and neutralize the perception of the uniform ground. Contrary to what other observers have said, I affirm that we can always influence the issue of the conflict by the voluntary direction of the attention. When we try to decipher the print, the letters remain dominant, at least, at the spot whither our attention is directed. When we attempt, on the contrary, to follow the lines or the contours of the engraving, these are the parts which dominate. I find even by directing the attention to any object feebly illuminated we may cause to disappear a much more intense object which is painted on the other retina. In this manner I am able to examine the details of structure of white paper, and to neglect a strongly marked design situated at the corresponding point of the other field. The antagonism does not rest, therefore, upon predominance or the oscillations of a sensation, but upon fixity, or the oscillations of the attention. There, perhaps, does not exist a phenomenon which lends itself better to the study of the causes capable of directing the attention. But it is very necessary to remark that the voluntary intention of seeing sometimes with one eye, sometimes with the other, is not sufficient. It is necessary to evoke a very vivid sensual representation of what one wishes to see for this object really to appear. When we abandon the representations to their natural course, we see that those oscillations are produced to which is given the name of antagonism. Very apparent objects then generally get the better, at least for a time, of the less luminous or less clear objects of the other field.

Moreover, when we place two glasses of different colors before our eyes, and then look at the same objects, there is manifested an antagonism of colors analogous to those of objects which causes sometimes one and sometimes the other of these colors to dominate in places; only after some time, when the vivacity of the colors has diminished in the sensation, on account of unilateral fatigue, and by the production of complementary accidental colors, we see a sort of resultant color, which is composed of the two primitive colors.

It is much more difficult to direct the attention exclusively to one of the colors than to one of the two designs adapted to produce antagonism; because we can direct the attention to a sensual impression permanently only when we constantly find there something new to discover. Nevertheless, we may facilitate the experiment by directing the attention to the designs or letters reflected by the side of the glass which covers the eye. These reflected images are white, nevertheless, to direct the attention to them is sufficient to make the color of the glass, before which they are found, appear at once in the perception.

*To be Continued.*

